# Tumor-Associated Macrophages in Pancreatic Ductal Adenocarcinoma: Origin, Polarization, Function, and Reprogramming

**DOI:** 10.3389/fcell.2020.607209

**Published:** 2021-01-11

**Authors:** Sen Yang, Qiaofei Liu, Quan Liao

**Affiliations:** Department of General Surgery, Peking Union Medical College Hospital, Peking Union Medical College, Chinese Academy of Medical Sciences, Beijing, China

**Keywords:** tumor-associated macrophages, pancreatic ductal adenocarcinoma, polarization, reprogramming, origin

## Abstract

Pancreatic ductal adenocarcinoma (PDAC) is a highly lethal malignancy. PDAC is only cured by surgical resection in its early stage, but there remains a relatively high possibility of recurrence. The development of PDAC is closely associated with the tumor microenvironment. Tumor-associated macrophages (TAMs) are one of the most abundant immune cell populations in the pancreatic tumor stroma. TAMs are inclined to M2 deviation in the tumor microenvironment, which promotes and supports tumor behaviors, including tumorigenesis, immune escape, metastasis, and chemotherapeutic resistance. Herein, we comprehensively reviewed the latest researches on the origin, polarization, functions, and reprogramming of TAMs in PDAC.

## Introduction

**FIGURE 1:**
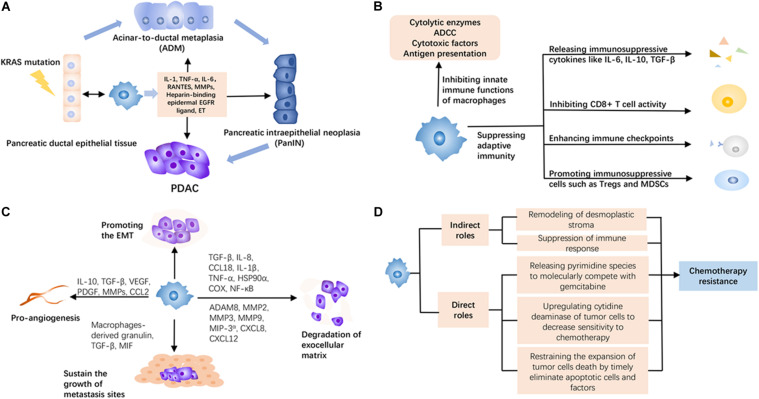
The roles of tumor-associated macrophages (TAMs) in pancreatic ductal adenocarcinoma (PDAC). **(A)** TAMs can produce more inflammatory stimulations to promote acinar-to-ductal metaplasia (ADM) and pancreatic intraepithelial neoplasia (PanIN) from normal tissue under KRAS mutation, thus accelerating the maturation of PDAC. **(B)** TAMs induce immunosuppression in the PDAC microenvironment *via* multiple ways, including releasing immunosuppressive cytokines, inhibiting the activity of tumor-infiltrating immune cells, and facilitating other inhibitory cells. **(C)** TAMs can promote the early metastasis of PDAC through proangiogenesis, promoting epithelial–mesenchymal transition (EMT), degrading the exocellular matrix, and sustaining the survival and growth of metastasis sites. **(D)** TAMs mediate chemotherapy resistance in an indirect and direct way. They create an adverse environment by remodeling desmoplastic stroma and suppressing immune response against the efficacy of chemotherapy. Meanwhile, they have a system of adaptation to reduce the attack of chemotherapeutic agents like releasing pyrimidine species to molecularly compete with gemcitabine, upregulating cytidine deaminase of tumor cells to decrease sensitivity to chemotherapy, and restraining the expansion of tumor cell death by timely eliminating apoptotic cells and factors.

**FIGURE 2:**
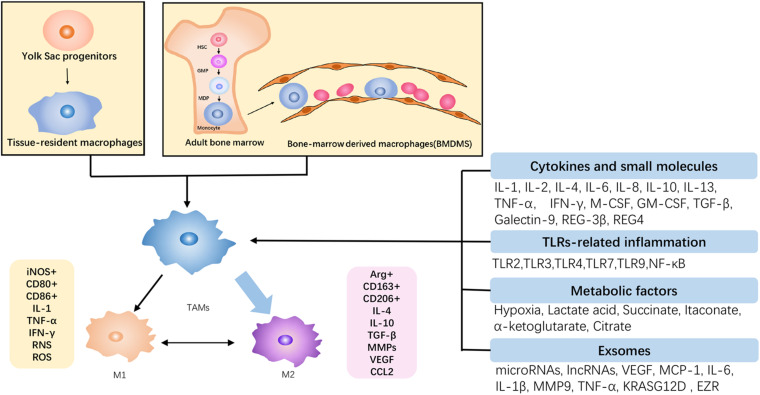
The origin and polarization of TAMs in PDAC. TAMs originated from the self-renewal of yolk sac progenitors and the recruitment of bone marrow-derived monocytes, and they are gradually differentiated into TAMs under tumor circumstance. Subject to tumor microenvironment, TAMs are predominantly orchestrated toward the M2 phenotype, which is protumoral, and there is a transformation between antitumoral M1 and M2. Accordingly, the increase of M2 infiltration contributes to the malignant growth of PDAC.

Pancreatic ductal adenocarcinoma takes a proportion of 85% in pancreatic cancer cases and is still one of the most malignant tumors with 5-year overall survival of less than 10% (Foucher et al., 2018). Fewer than 20% of patients are candidates for curative surgery since the overwhelming proportion of patients with PDAC have presented locally advanced or distant metastatic disease (Singhi et al., 2019). PDAC is a well-known inflammatory cancer. Distinctive to acute inflammations, immune activity in a tumor microenvironment is extremely repressive, and chronic substimulation by that immunity results in further growth and proliferation of tumor cells. The immune suppression in the tumor microenvironment enables tumor cells to escape from the immune surveillance and elimination by the antitumor immunity system, which plays a pivotal role in multiple solid tumors including PDAC (von Ahrens et al., 2017). Nevertheless, immune components in PDAC are complicated and an intricate cross talk connecting tumor cells and stromal cells leaves the single immune targets invalid in immunotherapy; therefore, it is imperative to find effective strategies to incite significant and extensive alteration in a pancreatic tumor microenvironment (Morrison et al., 2018; Bear et al., 2020). Macrophages in the tumor stroma, called TAMs, are one of the most abundant immune cell populations in the tumor microenvironment. TAMs can be differentiated into subsets with distinctive phenotypes and functions, which is called macrophage polarization. The traditional view offers a dichotomy concerning macrophage polarization, the M1 and M2 type. M1 macrophages are pro-inflammatory, while M2 is anti-inflammatory, which corresponds to the antitumor and protumor in the tumor microenvironment (Lankadasari et al., 2019). Although TAMs always have a dynamically changeable status in PDAC, they are inclined to M2 deviation with protumor effects, such as promoting tumorigenesis, forming the immunosuppression, accelerating metastasis, inducing chemotherapeutic resistance, and so on. According to the above, impeding M2 macrophage formation is of vital significance in hindering PDAC development, improving antitumor immunity and even clinical therapy. Currently, a growing body of reports on TAMs in PDAC has been published; herein, we comprehensively reviewed the origin, polarization, roles (Figure 1), and reprogramming (Figure 2) of TAMs in pancreatic cancer.

## The Role of TAMs in PDAC

### Promoting Tumorigenesis

Numerous researches on tumor evolution make the consistent opinion that maturation of PDAC requires a long-standing and persistent precancerous state. ADM and PanIN are acknowledged as indispensable precancerous processes. Acinar cells with high plasticity have undergone differentiation to a progenitor-like cell type with ductal characteristics under external stress, termed as ADM. Subsequently, cells following ADM in response to oncogenic signaling are precursors for PanIN lesions, which can further progress to PDAC (Storz, 2017). More than 90% of clinical PDAC cases have been detected as KRAS mutations, frequently occurring in the threshold of precancerous lesions, to accelerate the ADM and PanIN (Hong et al., 2012; Kamisawa et al., 2016). Featuring as fibrous inflammation incorporating immune cells, fibroblasts, extracellular matrix, vessels, and nerves, the initiation of PDAC depends on the intricate and consecutive interactions between epithelial cells and stroma (Zhang Y. et al., 2019). Macrophages are recruited and accumulated at the early stage of pancreatic precancerous lesions, which can be regarded as one of the earliest immune cell responses (Liu et al., 2016). Bidirectional signaling between the epithelium and macrophages has been recently noted. Epithelial KRAS promoted protumorigenic expression patterns in macrophages which in turn augmented cancerous phenotypes in the epithelium (Bishehsari et al., 2018). In pancreatic tumorigenesis, oncogenic KRAS augment the sensitivity to carcinogenic stimulation by multiple steps and offer an incentive to the accomplishment of ADM and the formation of PanIN, which are engined by various stress, such as inflammation (Carrer et al., 2019; Nishikawa et al., 2019). Among the related inflammatory pathways, the activation of STAT3 serves as a significant tumorigenic pathway, actuated by macrophage-derived pro-inflammatory cytokines, such as IL-6 and IL-10, increasing the sensitivity of acinar cells to reprogram under inflammatory stimuli. Activation of the JAK–STAT3 pathway requires the facilitation of YAP1 and TAZ signaling (Gruber et al., 2016). The ADM induction has also underlined another two macrophage-derived pro-inflammatory cytokines, RANTES (CCL5) and TNF-α, *via* a NF-κB-dependent manner and PI3K/Akt/IKK signaling pathway, engaging in survival, proliferation, and degradation of the extracellular matrix (Huang et al., 2015). The ET axis in the ductal and stromal cells has been proposed to have a potential role in the initiation and progression of PDAC at the background of mutant KRAS, promoted by various inflammatory stimuli in the environment. Especially, ET-1 binds to its dual receptors, ETAR and ETBR, interconnecting the interactions among epithelial cells, TAMs, CAFs, and other immune cells (Gupta et al., 2020). Besides, TAMs predominantly enhance the expression of heparin-binding EGFR ligand in pre-neoplastic lesions to facilitate ADM (Kumar et al., 2015). Fibrous inflammation activates MMPs to remodel the tumor microenvironment and MMP inhibitors remarkably decelerate pancreatitis-induced ADM (Liou et al., 2013). Furthermore, TAM-derived IL-6 induces the phosphorylation of PDPK1-mediated PGK1 threonine (T) 243 in tumor cells, to facilitate a PGK1-catalyzed reaction toward glycolysis by altering substrate affinity, which upholds PDAC initiation in a metabolic regulation way. Neutralization of macrophage-derived IL-6 or inhibition of PGK1 T243 phosphorylation or PDPK1 in tumor cells markedly abrogates macrophage-promoted glycolysis, proliferation, and tumorigenesis (Zhang Y. et al., 2018). It is remarkably observed that the elimination of TAM populations in pancreatic lesions by the immunomodulatory agent pomalidomide successfully turns the microenvironment from immunosuppressive to immune-responsive, which prevents ADM transformation and PanIN formation (Zhang Y. et al., 2017; Bastea et al., 2019). The mutilation of NSAIDs has been evidenced as effective in tumor precaution, such as aspirin, celecoxib, diclofenac, diflunisal, and ibuprofen. On the contrary, the result from several large cohort studies has refuted the view that regular use of NSAIDs was not associated with future risk of pancreatic cancer in participants (Khalaf et al., 2018). However, it should be noted that regular aspirin application ablated the risk of PDAC among participants with higher systemic inflammation induced by diabetes and hyperglycemia in contrast to local inflammation. This put forward a viewpoint into inflammatory response in the tumor microenvironment.

### Immune Escape

As the front line of innate immune defense, macrophages play significant roles in tumor immunity, including the direct tumor-killing effect by releasing cytolytic enzymes, triggering ADCC *via* surface Fc receptors on the macrophages, secreting cytotoxic factors such as TNF, and presenting of tumor antigens to activate specific T-cell immune response. In the initial phase, macrophages can implement their innate immune functions to eliminate tumor cells, while these direct roles will be covered by tumor cells. For instance, phagocytosis is impaired resulting from tumor cells hijacking “not-eat me” labeling such as CD47-SIRPα to evade the attack of macrophages (Michaels et al., 2018). Indeed, phagocytosis depends on the promotion of the homeobox protein VentX *via* regulating a series of the signaling cascade, which was previously correlated with the immune function of macrophages and the M1 orientation of TAMs (Wu et al., 2011; Le et al., 2018, 2020). Additionally, tumoricidal factors produced by activated macrophages such as TNF-α and NO can be invalidated. Tumor cells can inhibit NF-κB and further downregulate its downstream genes *Tnf* and *iNOS* by upregulating the expression of GDF-15, ultimately leading to a reverse hampering to the production of these cytokines (Ratnam et al., 2017). Such tumor immune microenvironment hinders macrophages to implement their immune surveillance, in a way that directly weakens the immune system.

One crucial mechanism concerning the PDAC microenvironment is relying on a panel of immunosuppressive cytokines produced by M2-type TAMs, containing IL-10, TGF-β, IL-6, PGE, CCL2, CCL17, CCL20, and others, which inhibit immune cell activity like CD8^+^ T cells and convert the inflammatory response into calmness (Principe et al., 2016; Daley et al., 2017; Eriksson et al., 2019). Immune checkpoints refer to the co-stimulatory and inhibitory signals of immune cells capable of curbing immune response, frequently adopted by tumor cells to suppress surrounding immune elimination (Martinez-Bosch et al., 2018). TAMs aggravate the signaling of immune checkpoints, such as the increased secretion of CTLA-4, PD-L1, which undermined immune recognition by T cells and enhanced immune tolerance (Mantovani et al., 2017). On the other hand, the upregulation of CTLA4 and PD-L1 on T cells and tumor cells also regresses the recruitment and infiltration of TAMs (Zhang Y. et al., 2017). Immune checkpoints on macrophages can likewise lessen immune functions of immune cells incorporating themselves. Macrophage-expressed PD-1 is influential to its phagocytic potency against tumor cells, to induce the immune tolerance of innate and adaptive immunity (Gordon et al., 2017). Other immunosuppressive components have functional connections with TAMs, such as Treg, their immunosuppressive induction dependent on TAM assistance. TAMs participate in the differentiation and maturation of Treg cells from CD4^+^ T lymphocytes, thus enriching the Treg population. The study of Zhou et al. (2018) has found that TAM-derived exosomes carry out multiple miRNAs including miR-29a-3p and miR-21-5 to stimulate T cells, causing a rise in the Treg differentiation ratio *via* STAT3 activation. Certainly, immune checkpoints of T cells such as PD1/PD-L1 subject to TAMs equally induce the differentiation and functional maturation of Treg cells (Seo and Pillarisetty, 2017). In summary, the immunosuppressive roles of TAMs consist of producing inhibitory cytokines, diminishing the effector of tumor-infiltrating lymphocytes, and promoting immunosuppressive cells.

### Metastasis

Tumor-associated macrophages infiltration accelerates tumor growth and metastasis, emerging as malignant outcomes and adverse prognosis (Lankadasari et al., 2019). PDAC growth relies on the delivery of nutrients and oxygen, owing to the aerobic glycolysis of tumor cells. Based on the demand, angiogenesis is a critical pathological behavior for tumor cells to exempt from scant nutrients and oxygen (Daniel et al., 2019). A visible aggregation of massive macrophages was reported to occur in the hypoxic area of PDAC and a large proportion of them surrounds intertumoral vessels, implicating the essential association between TAMs and angiogenesis. However, stromal pressure has been increasingly elevated as the expansion of tumor entity, resulting in vascular collapse up to an absence of large diameter vessels in pancreatic cancer (Li S. et al., 2019). Despite the shortage of vessels in PDAC, a high level of pro-angiogenetic factors like VEGFA promotes the increased risk of early metastasis (Rigamonti et al., 2014). Exuberant angiogenesis exerts the prometastasis in PDAC as common as other solid tumors, which remains the therapeutic target. Tumor metastasis depends on the stromal degradation and the loss of intercellular connections, which can be realized by TAMs *via* secreting matrix proteins and proteases such as serine proteases, MMPs, and cathepsins (Cui et al., 2016). Especially, pancreatic tumor cell migration rate can be enhanced through TAMs regulating the expression of ADAM8 and MMP9, conductive to breach the basement membrane and accrete the invasiveness (Puolakkainen et al., 2014). Notably, MMP9 is an essential factor, which enhances the degradation of the extracellular matrix protein laminin, as a major component of blood vessels, which directly manipulates the disintegration of the vessel wall for extravasation (Knapinska et al., 2017; Tekin et al., 2020). Likewise, MMP9 overproduction can be induced by CAF-derived IL-33 *via* the IL-33–ST2–NF-κB–MMP9 axis (Andersson et al., 2018). Additionally, several chemokine signals help tumor cells to penetrate blood vessels. These chemokines like CXCL8 and CXCL12 collaboratively aggravate invasion and angiogenesis in pancreatic cancer, *via* their corresponding receptors, CXCR2 and CXCR4 (Matsuo et al., 2009). Meanwhile, these signals could also drive macrophages to M2 polarization (Pausch et al., 2020; Zhang et al., 2020). Moreover, TAM-induced inflammation also promotes EMT in PDAC, which is defined as a phenotypic switch from epithelial to mesenchymal phenotype cell through gradually vanishing cell polarity and intercellular connections, consequently enhancing tumor cell migration and invasion. Pancreatic cancer cells following co-culture with M2 macrophages showed the upregulation of mesenchymal markers vimentin and Snail coupled with downregulating the epithelial marker E-cadherin (Liu et al., 2013). However, EMT in PDAC is irrespective of TAM polarization, and both pro- and antitumor phenotype can contribute to the conversion (Helm et al., 2014). Apart from these M2-type cytokines such as TGF-β, IL-8, and CCL18 (Meng et al., 2015; Chen S. J. et al., 2018), emerging evidence has revealed that the pro-inflammatory activation by TAMs is capable of EMT promotion, such as the release of IL-1β and TNF-α, and the activation of COX and NF-κB (Chen et al., 2019). Interestingly, pancreatic cancer cells after EMT can secrete HSP90α to mediate M2 polarization, while M2 macrophages overproducing HSP90α actuate pancreatic tumorigenesis. It is like a reciprocal loop due to the coordination of tumor microenvironment constitution (Fan et al., 2019). Loose cells and leaking blood vessels allow free tumor cells into the bloodstream and spread throughout distant organs. The colonization of metastatic tumor cells at distant organs critically requires the support of surrounding non-cancerous stromal components, especially TAMs appearing as a surge in the metastatic site (Costa-Silva et al., 2015). Nielsen et al. (2016) have demonstrated that a specific granulin by TAMs can activate stellate cells into αSMA^+^ myofibroblasts with a high yield of periostin, sustaining the survival of metastatic cells. Simultaneously, normal resident macrophages can be educated by PDAC cells to protumoral and prometastatic phenotype. Activating TGF-β signaling of liver-resident macrophages is induced through PDAC cell-derived exosomes, thus constituting the pre-metastatic niche by activating HSCs and remodeling ECM and exosome-derived migration inhibitory factor (MIF) as a well-known mediator of liver inflammation and fibrosis (Costa-Silva et al., 2015).

### Chemotherapy Resistance

Clonal selection for a resistant population belongs to one of the numerous tumor cell-autonomous responses, for their adaptation to a stressful environment. TAMs promote resistance, while gemcitabine treatment in turn attracts abundant TAM infiltration (Liu et al., 2016; Liu Q. et al., 2020). Pharmacological depletion of TAMs overthrows chemoresistance like gemcitabine, leading to an obvious improvement of PDAC therapy (Céspedes et al., 2016). Dense stroma is conceived as a key factor to hinder medicine delivery, and TAMs promote stroma formation by activating cancer-related fibroblasts (Zhang D. et al., 2018). The existence of TAMs influences antitumoral drug delivery, biophysically creating an impenetrable medium to hamper the pharmacokinetic motion of gemcitabine (Buchholz et al., 2020). TAMs directly induce the resistance of PDAC to gemcitabine and the apoptosis of PDAC cells has been minimized upon incubation with a TAM-conditioned medium (Xian et al., 2017). The present understanding of TAMs to chemotherapy resistance remains not illuminated. TAMs are capable of the upregulation of cytidine deaminase to decrease their sensitivity to gemcitabine by accelerating the metabolism of gemcitabine (Binenbaum et al., 2018). Meanwhile, a sphere of pyrimidine species is liberated by TAMs to molecularly compete with gemcitabine, thus influencing drug uptake and metabolism and largely reducing the efficacy of chemotherapy (Halbrook et al., 2019). The immunosuppressive cytokine TGF-β1 secreted by TAMs can upregulate CTGF and HMGB1 by virtue of regressing Gfi-1 expression (Xian et al., 2017). The hypothesis has been evidenced by D’Errico et al. (2019) that the timely phagocytosis and clearance of TAMs to apoptotic cells and related factors prevent further expansion of cellular apoptosis and contribute to chemoresistance to gemcitabine, which is associated with the 14-3-3ζ/Axl signaling. In research on hepatic carcinoma, TREM-1 expressing TAMs exhibited as the fundamental bridge to enhance CCR6^+^ Foxp3^+^ Treg accumulation, which disabled targeting PD-1 therapy, while the blockade on TREM-1^+^ TAMs revived the therapeutic activity (Wu et al., 2019). It provides us with a novel insight to consider therapeutic resistance by targeting TREM-1^+^ TAMs in pancreatic cancer. Although the mechanism concerning TAMs inducing tumor chemotherapy resistance is still obscure, TAM amounts are paralleling with chemotherapy resistance, which implicates the significance of TAMs as a target to chemotherapy resistance.

## The Origin and Recruitment of TAMs in PDAC

Traditional researches on the monocyte–macrophage system hold the dichotomous concept, tissue-resident macrophages and BMDMs. Tissue-resident macrophages have a dual identity in disease development: they trigger an inflammatory response by PRRs and recruit inflammatory cells to elicit the inflammation cascade; it delineates the limit of inflammation, tranquilizes the immune system, and repairs damaged tissues (Ginhoux and Guilliams, 2016). Nonetheless, tissue-resident macrophages cannot offset consumption and acquire rapid replenishing by BMDMs to sustain pathogen challenge and energize inflammation until further explosion (Zhu et al., 2017). Macrophages in the tumor microenvironment are heterogeneous and its origin has been controversial until now. It has been historically insisted that mature tissue-resident macrophages are merely from BMDMs, whereas accumulative advances have proposed the yolk sac progenitors seeding tissues during the fetal and embryonal period as the major origin of such macrophages in healthy tissues, which acclaims its self-renewal rather than replenishment of peripheral circulation monocytes (Ginhoux et al., 2016; Mantovani et al., 2017). Zhu et al. (2017) found that TAMs in PDAC do not always originate from HSCs, but can also proliferate and differentiate from self-renewing embryonic precursors, which casts doubt about the immunological dogma of TAM derived from circulating monocytes. Tracing the origin of tumor macrophages has inspired the study of the phenotype and function of multisource macrophages. The accumulation of Ly6C^hi^ circulating inflammatory monocytes in glioma contributes to an increase in tumor incidence and shortened survival expectancy, while inherent macrophages and microglia do not (Chen et al., 2017). As for PDAC tumorigenesis, BMDMs focused on antigen presentation and self-renewal TAMs are inclined to a profibrotic phenotype, implicating their role in the constitution of the extracellular matrix (Zhu et al., 2017). Furthermore, macrophage recruitment is a complex multistage process with the involvement of multiple cytokines, signal transmission, and cellular interaction. Chemoattractants like CCL2, CCL5, VEGF, and CSF-1 attract circulating monocytes in a gradient of concentration (Nielsen and Schmid, 2017). The CCL2/CCR2 axis particularly displays irreplaceability to recruit macrophages from bone marrow to tumor lesion. In post-resection patients of PDAC, CCL2/CCR2 chemokine signals can be prognostic of reduced survival (Sanford et al., 2013). The blockade of the CCL2/CCR2 axis has been employed in PDAC researches for reducing the TAM population, which accomplishes significant tumor regression (Long et al., 2016). A recent study has revealed a novel approach to aggregating macrophages: the neurotransmitter receptor unit, gamma-aminobutyric acid type A receptor pi subunit (GABRP), can ultimately induce the CXCL5 and CCL20 to mediate the recruitment of TAMs in pancreatic cancer *via* the activation NF-κB signaling by interacting with KCNN4 to trigger Ca2^+^ entry (Jiang et al., 2019).

## Inflammatory Cytokines, CSFs, and Soluble Small Molecules on TAM Polarization

Chronic inflammatory cytokines have a positive relationship with poor prognosis in PDAC patients, and these cytokines coordinating with immunosuppressive cells induce immune suppression in the local surrounding of cancers (Farajzadeh Valilou et al., 2018; Feng L. et al., 2018). In the conventional polarization theory, alternatively activated macrophages depend on IL-4 and IL-13 through their share receptor IL-4Rα, and then actuate JAK/STAT6 to ignite marker gene explication. Their productions fluctuate in different phases. In the precancerous phase PanIN, those mutant cells have taken the major responsibility of secreting them (Liou et al., 2017). Pancreatic stellate cells (PSCs) become the sustainable and principal supplier of IL-4/IL-13, attributing to the histopathological feature of an ample fibrous matrix (Xue et al., 2015). Targeting the SH2 domain of STAT6 by phosphopeptide mimetic PM37 enables to repress IL4/IL13-mediated STAT6 phosphorylation and downregulates M2 polarization markers (Rahal et al., 2018). Besides, the activation of IL-4/IL-13 signaling can be managed by the adaptive factor c-MYC, which relays the impulse to their downstream mediators of transcription-6 and PPARγ to undergo M2 reprogramming, with the elevated expression of VEGF, MMP9, HIF-1α, and TGF-β (Pello et al., 2012). Another typical member IL-10, as a product of M2 macrophages, can also act on IL-10R of TAMs in turn, to enhance M2-like gene appearance by the JNK/STAT3 pathway, which forms a feedback loop (Fu et al., 2017). IL-6 is a cytokine with both pro-inflammatory and anti-inflammatory roles, diversely performing in different diseases. IL-6-induced macrophage seems to appear as an immune-tolerant type *via* equally activating JAK/STAT3 pathway, and co-stimulation with CSF1 can induce the expression of Arg1 involving the PPARγ-dependent transcriptional activation of HIF-2α (Wang Q. et al., 2018). Recently, IL-8 has been found to have a novel role in gemcitabine-induced infiltration of macrophages, which suggests the significant role of IL-8 in TAM-related chemotherapy resistance (Deshmukh et al., 2018). Acute inflammatory cytokines are previously reckoned as M1-like activators, such as IL-1β, TNF-α, and IFNγ, which are exemplified classically in multiple tumor models. In acute inflammation, differentiation of CD4^+^ T cells toward Th1 and Th17 cells relies on IL-1β signaling, favorable to pathogen elimination (Bent et al., 2018). However, it is in a subactivated state in chronic inflammation or tumor, promoting tumorigenesis through its downstream signals and cytokines and creating M2 tendency. IL-1β can also be produced by M2 macrophages and M2-derived IL-1β enhances the synthesis of HIF-1α through cyclooxygenase-2, ultimately facilitating EMT and metastasis (Zhang J. et al., 2018). TNF-α as a typical representative of pro-inflammatory cytokines carries the core duty in acute inflammation by triggering cell apoptosis and activating the NF-κB signaling pathway. M2-type macrophages and tumor cells are qualified to release TNF-α in a low concentration (Farajzadeh Valilou et al., 2018). TNF-α inducing cell death leaves a massive cell debris to the tumor microenvironment that can be recognized by macrophages and thus actuated the orientation toward M2 type (Chen et al., 2019). As a soluble dimeric cytokine in charge of tumor immune surveillance and cytotoxicity, IFNγ endows macrophages polarized toward an M1 state of enhancing pro-inflammatory reaction and resisting to immune tolerance (Ivashkiv, 2018). However, co-stimulation of IFNγ and IL-21 provides conditions for protumorigenic M2 macrophages, which further expedite the tumor progression (Chen et al., 2016). Undoubtedly, adequate supplementation of these cytokines is advantageous to M1 deviation. Recent studies have reported that elevation of TNF-α and IL-2 in tissues by transfecting oncolytic adenovirus loaded with TNF-α and IL-2 sequence into T cells modulates host tumor immune status and orchestrates TAMs toward the M1 type (Watanabe et al., 2018). The remarkable antitumor efficacy of exogenous IFNγ administration has also been witnessed in pancreatic cancer, coupled with the reversing ratio of M1/M2 (Zhang et al., 2020). Accordingly, their specific efficacy is concentration dependent, with double roles for tumor progression. Phenotypes of TAMs are synthetically determined by assorted stimulations rather than one certain way, and the transformation from M1 to M2 implicates a dynamic variation of the macrophage phenotype, which is fundamentally attributed to the overall ambiance of the tumor microenvironment.

Colony-stimulating factors have decisive impacts on the cytogenesis of HSCs at different developmental stages, as essential stimulators for the generation of blood cells (Metcalf, 2013). Notably, CSF-1 (M-CSF) and CSF-2 (GM-CSF) are two critical cytokines to regulate the circulated reservoir of monocytes/macrophages (Jeannin et al., 2018), which shoulder different functions of body needs. CSF-1 has been reckoned as an adverse prognostic factor, and its serum level keeps the same pace with tumor progression in multiple tumor models (Ławicki et al., 2016). CSF-1 is engaged in the development, morphology, survival, and function of TAMs, under a stable and persistent production of a relatively high level, which is recognized by CSF-1R to activate molecule adaptors PI3K and Grb2, to phosphorylate the PI3K/Akt and MAPK pathways (Bencheikh et al., 2019; Evrard et al., 2019). Macrophages stimulated by CSF-1 exhibit immunoregulatory properties, tending to repress inflammation, and repair damaged tissue. Thus, they hamper the tumor-killing immune effect during tumor progression, significantly overproducing IL-10 while decreasing IL-12 (Jeannin et al., 2018). A recent study has revealed that CSF-1 can induce macrophages to express granulin to exclude cytotoxic CD8^+^ T cells from stroma, while genetic depletion of granulin impedes the formation of stroma and increasingly restores antitumor immunity (Quaranta et al., 2018). TREM-1^+^ macrophages inducing chemotherapy resistance may be in a CSF-1-dependent antitumor mechanism (Shen and Sigalov, 2017). Furthermore, CSF-1R blockade significantly undermines the population of TAMs to a large degree, thereby reducing/diminishing M2-like TAM infiltration, but it has no definite effect on tumor cells (Pyonteck et al., 2013). Anti-CSF-1 therapy alters the tumor immune microenvironment by removing macrophages and reducing M2 infiltration, which is a useful therapeutic target in pancreatic cancer treatment. IL-34 has been identified as an alternative ligand for CSF-1R, but IL-34-activated macrophages produce more IL-10 and CCL17, compared to the mode of CSF-1 action (Lin et al., 2008; Boulakirba et al., 2018). Distinctive to CSF-1, CSF-2 has a lower basal circulation level under homeostasis, but its level surges rapidly during infection or inflammation (Zhan et al., 2019). CSF-2 is the product of activated state under pathological conditions which can bring about the prosperity of the macrophage population, and thus, serves as a potent immune enhancer in inflammation or infection diseases (Wang et al., 2016; Grasse et al., 2018; Venet and Monneret, 2018). Similarly, boosting immunity by CSF-2 can be successfully replicated in some neoplastic diseases, as an immune adjuvant for promoting antitumor immunity (Oh et al., 2017; Yan et al., 2017). Nevertheless, CSF-2 analogous to pro-inflammatory cytokines can promote epithelial and stromal interactions to accelerate tumorigenesis (Waghray et al., 2016). A remarkable rise in CSF-2 has been measured after exposure to tobacco-specific nitrosamine 4-(methylnitrosamino)-1-(3-pyridyl)-1-butanone (NNK), which effectively augments the level of cyclic AMP response element-binding protein (CREB) to accelerate pancreatic tumorigenesis (Srinivasan et al., 2018). Likewise, chemotherapy resistance is induced by CSF-2 *via* mediating the differentiation of monocytes into MDSCs, as well as hampering the tumoricidal effect of T cells (Takeuchi et al., 2015). Recent cohort analysis has proposed the highest elevated level of TGF-β1 and CSF-2 following gemcitabine treatment, suggesting their contributions to immunosuppression and chemotherapy resistance. The subsequent dual blockade of two cytokines has improved the chemotherapeutic efficacy by hampering M2-polarized TAMs and prospering CD8^+^ T cells (Liu Q. et al., 2020). Comparative research on the respective macrophage-associated functions of CSF-1 and CSF-2 has been conducted, concluding that CSF-1–macrophages are preferentially converted from Ly6C^hi^ monocytes into MHC-II^hi^ TAMs, while GM-CSF only fine-tunes the MHC-II^hi^ phenotype without significantly affecting the TAM populations (Van Overmeire et al., 2016). Although the directing action of CSF-2 is not significant, the immunosuppressive tumor microenvironment created by CSF-2 is deeply influential to TAM reprogramming. Pro-inflammatory macrophages activated and summoned by CSF-2 can be converted to M2-like macrophages, educated by immunosuppressive circumstances (Zhan et al., 2019). The immune function of CSF-2 is likely dose dependent. A relatively high level of endogenous or exogenous CSF-2 contributes to the amplification of M2-like suppressor cells, while a low dose of CSF-2 acts as an immune adjuvant (Parmiani et al., 2007). Despite CSF-1 and CSF-2 distinctively influencing macrophages, the unique properties of the tumor immune microenvironment lead tumor-infiltrating macrophages toward the protumoral phenotype. To sum up, the role of CSFs mainly focuses on promoting the proliferation, survival, and maturation of monocytes/macrophages, and their abundance provides basic conditions for macrophage polarization.

Lectin exhibits a specific affinity toward specific glycan structures to form a relatively strong complex, which is detected in multiple tumors and a useful detective tool for cancer diagnosis (Silva, 2019). It undertakes diversified functions such as signal transmission during the ontogenetic process, which plays a crucial role in PDAC progression. Galectin-9, a β-galactoside-binding lectin, switches the protumor M2 polarization leading to suppressed T-cell cytokine secretion, and its serum concentration can discriminate PDAC and serves as a prognostic for stage IV patients (Seifert et al., 2020). Macrophages highly expressing dectin 1, as the receptor of galectin-9, and their activation can reprogram tolerogenic macrophages to suppress adaptive immunity (Daley et al., 2017). The potential risk factor for PDAC regenerating islet-derived 3β (REG3β) acquires an increase in serum and pancreatic juice of PDAC patients. The presence of REG3β triggers M2 polarization through the activation of the STAT3 signaling pathway, while its deficiency led to a decrease in the M2/M1 ratio in the tumor area (Folch-Puy, 2013). Another REG family member, REG4, secreted by PDAC cells, promotes TAM polarization to M2, *via* a signaling transmission of the EGFR/AKT/CREB pathway (Ma et al., 2016). These small molecules secreted from tumor cells modulate the microenvironment which is advantageous to the survival and progression of tumor cells. These signaling molecules facilitate the communication between tumor cells and stroma while complicating the network of cell cross talk, which renders it harder to break the stabilization of the tumor microenvironment. The underlying network constituted by assorted cytokines and small molecules leaves an open question, and its potential influence on tumor requires further investigation.

## TLRs-Related Inflammation on TAM Polarization

Cancer-related inflammation as the seventh hallmark of cancer is categorized as unresolved inflammation. TLRs belonging to PRRs are extensively expressed on dendritic cells, macrophages, endothelial cells, and tumor cells. The non-specific recognition of TLRs consolidates the initial immune defense with multiple agonists to trigger an inflammatory response. The double face of TLRs in tumor development includes anti- and protumoral roles. On one aspect, the recognition of tumor-derived antigens reignites the agitation of innate and adaptive immunity for the enforcement of immune surveillance. Recent advances in TLR-related studies have suggested that TLR activation may indeed represent a relevant antitumor pathway, allowing to convert immune tolerance to antitumor immune response (Pradere et al., 2014). Moreover, the high expression of TLR9 in PDAC implies prolonged survival and superior prognosis (Leppänen et al., 2017). However, chronic inflammation mediated by TLRs is undoubtedly competent to incite tumorigenesis, which is exactly proved by an increased risk of carcinogenesis in patients with chronic inflammation, indicated by the relationship of hepatocellular carcinoma and chronic hepatitis, colorectal cancer and inflammatory bowel disease, gastric cancer, and *H*elicobacter *pylori* infections (Diakos et al., 2014). Certainly, systemic or local chronic inflammation aggravates the risk of PDAC (Padoan et al., 2019). T2DM and chronic pancreatitis have been identified as the risk factors for pancreatic tumorigenesis (Andersen et al., 2017). Emerging evidence has revealed the conservative role of TLRs in pancreatic cancer progression. In precursor lesions of early pancreatic cancer, PanIN, the aberrant expression of TLR2, TLR4, and TLR9 was also detected in PDAC (Leppänen et al., 2018). Constitutive activation of IRAK4, the downstream effector of TLRs, predicts poor prognosis and chemoresistance in PDAC (Zhang D. et al., 2017). Subsequently, the stimulation of TLRs can actuate some adverse protumoral signaling pathways, such as TLR2, TLR4, and TLR9, which activate signaling pathways and anti-apoptotic Bcl-xL expression in terms of tumor cell activation and proliferation in pancreatic cancer (Grimmig et al., 2016). Likewise, TLR7/TLR8 overexpressing pancreatic tumor cells promote the expression of NF-κB and COX-2, increasing cancer cell proliferation and reducing chemosensitivity (Grimmig et al., 2015). The influences of the TLR signaling pathway activations are pleiotropic in cancer progression, one key regulation of TAMs infiltrating in the stroma of PDAC. TLR activation switches on macrophage polarization to portray the image of a macrophage phenotype. A previous study has reported that macrophages exposed to TLR7/TLR9 ligands deviated into the M2 state as well as decreased antigen presentation (Celhar et al., 2016). As far as the related mechanisms are concerned, TLR-mediated macrophage differentiation requires an intricate regulatory network to actuate marker gene expression. A recent study has proposed that the CD14 antigen, a glycosyl-phosphatidylinositol (GPI)-linked glycoprotein, may incorporate in the signal transmission of TLR-mediated macrophage polarization (Cheah et al., 2015). Additionally, the phenotype of TAMs likewise complies with mitochondrial dynamics. FAM73b, a mitochondrial outer membrane protein, is involved in TLR-regulated mitochondrial morphology from fusion to fission, mediating M1 conversion *via* the CHIP–IRF1 axis (Gao et al., 2017). STAT3 as the downstream effector can be stimulated by TLR activations, which is imperative to M2 orchestration. Tumor cell-released autophagosomes (TRAPs), a type of LC3-II + double-membrane exocellular vesicles, depend on TLR4-mediated MyD88–p38–STAT3 signaling (Wen et al., 2018). Resistin with a significant role in insulin resistance has been reported to be secreted by macrophages and it activates the STAT3 signaling pathway by TLR4 and CAP1, which can aggravate cancer progression (Zhang M. et al., 2019). Moreover, tumor-secreted cathepsin K (CTSK) could stimulate TLR4 to switch to M2 polarization relying on the mTOR signaling activation (Li R. et al., 2019). Profiling experiments showed that the transcription factor CUX1 is an important modulator of the TAM phenotype by participating in NF-κB inflammatory signal activation (Kühnemuth et al., 2015).

Cellular debris and contents can be recognized by the corresponding PRRs on immune cells to trigger cancer-related inflammation, which is identified as DAMPs including HMGB1, hot shock protein, hyaluronan, fibrinogen, nucleic acid fragments, and so on (Patidar et al., 2018; Miller-Ocuin et al., 2019; Zhang B. et al., 2019). After NACRT, DAMP overproduction acquired a purposeful immune response and longer survival of PDAC patients (Murakami et al., 2017). The immune-activating capability of DAMPs may be applied to cancer therapy as a vaccine approach, to increase the probability of evoking broader and all-embracing cytotoxic and memory T-cell responses (Lybaert et al., 2018). This reflects the antitumor effect of the early inflammatory response in tumors. However, sustained activation of the immune system has an opposite effect and the restraining of DAMPs is beneficial from a long-standing perspective. Using nucleic acid-binding polymers (NABPs) to reduce nucleic acid-mediated TLR activation can combat pancreatic cancer growth and metastasis (Naqvi et al., 2018). Moreover, although the pancreas is not exposed to massive microbes like the gut, whose lesions are closely associated with microbial lives, recent advances have offered a bright insight into the role of microorganisms in pancreatic tumors. The oncogenic role of microbial dysbiosis has been acknowledged in pancreatic cancer, especially influential to immune status and contracture. Microbiomes affect the development of pancreatic cancer, including inflammation and immunomodulation *via* innate and adaptive immunity (Wang et al., 2019; Wei et al., 2019). Depletion of the gut microbiome led to a series of alterations in the tumor immune microenvironment, incorporating the diminution of MDSC infiltration and the reorientation toward the M1-like phenotype and promoting the tumoricidal effects of T cells. It is possible in a TLR-dependent manner, and the absence of TLR-related signals makes it invalid to induce immunosuppressive macrophages by PDAC-bacterial extracts (Pushalkar et al., 2018). According to the above, TLR activation strikes the immune system by triggering an inflammatory response, but it will be ultimately converted to an immunosuppressive type, under the coordination of tumor cells and anti-inflammatory regulation of the body. The dual outcome of TLRs in PDAC can be witnessed: early antitumor and late protumor. The suggested application of TLR activation and inactivation targeting their different functional phases is an effective and promising strategy during PDAC therapy. Given the adaptability and conversion of long-term TLR stimulation, whether it will promote the development of PDAC remains a question, which requires more profound investigations.

## Metabolic Factors on TAM Polarization

The unique metabolism characteristics are one of the seven hallmarks of cancer. In particular, the tumor microenvironment is famous for the deficiency of oxygen supply and nutrients, which render settling cells to alter their metabolic methods. Changes in metabolic conditions regulate cellular expression of some characteristic genes to adapt to external stress, which is known as metabolic reprogramming. Macrophages in the tumor microenvironment indeed undergo metabolic reprogramming, with preferential amplification of adaptive genes. Generally, differentiation to the M2 phenotype is conducive to their survival in such hypoxic-, acid-, and glucose-deficient pancreatic cancer stroma. The environmental factors can guide TAMs toward its survivable differentiation. Hypoxia inducing cellular variation is a research hotspot in the recent medical domain, and hypoxia commonly occurs in many solid tumors and mediates the biological behavior of tumor cells (Semenza, 2012; Moreno Roig et al., 2018). Previous data have revealed a decrease in tissue partial oxygen pressure in PDAC, with median pO_2_ 0–5.3 mmHg compared to surrounding normal tissues at pO_2_ 24.3–92.7 mmHg (Koong et al., 2000). Considerable reviews have summarized the role of hypoxia in PDAC progression and proposed the significance of targeting hypoxia (Semenza, 2012; Daniel et al., 2019). The inhibition of hypoxia synergistically reinforced the tumoricidal efficacy of gemcitabine, as well as significantly prolonged the survival in KPC mice after tumor resection, which indicated a series of stromal alterations, incorporating increased vasculature and decreased fibrillar collagen, and the infiltration of immune-responsive macrophages and neutrophils (Miller et al., 2015). It should be affirmed that hypoxia affects dynamic alterations of macrophages in the tumor microenvironment. Macrophages in hypoxic areas show a more dominant alternative activation, which might illuminate a positive effect of tumor hypoxia on the M2 tendency (Wang X. et al., 2018). The gene expression profile of hypoxic macrophages varies including low expression of MHC-II, as well as strengthened hypoxic adaptations such as VEGFA, LDHA, and uPAR (Henze and Mazzone, 2016; Chen X. et al., 2018). Hypoxia recruits TAM precursors from bone marrow to tumor lesion, through multiple mechanisms such as releasing chemoattractants like CCL2, CCL5, CXCL12, VEGFA, and endothelin and liberating some cellular contents like DAMPs from the hypoxic dying cell (Li et al., 2016). The stabilization of hypoxia-inducible factors HIF-1α and HIF-2α has been detected in hypoxic macrophages of PDAC as tumor progression (Talks et al., 2000). HIF-1α has a more dominating part of hypoxia-inducing TAM polarization than HIF-2α. External hypoxic stimulations trigger its subsequent signaling molecules by inducing changes of HIF-1α, commonly like PI3Kγ, Akt, mTOR, c-myc, and so on, translationally and transcriptionally modulating marker genes (Attri et al., 2018). Certainly, Th1 and Th2 cytokines are involved in the stabilization of HIF-1α, which aggregately regulates the fate of macrophages (Riera-Domingo et al., 2020). Nevertheless, its role in tumor remains to correlate with malignancy and poor prognosis, resulting from a protumoral property of hypoxic macrophages. Ren et al. (2018) have found that miR-301a-3p-rich exosomes by tumor cells trigger HIF-1α stabilization to polarize macrophages to the M2 type, to promote malignant behaviors of PDAC. On the contrary, the irradiated tumors after low-dose radiation manifest the observation of TAMs toward an M1 phenotype due to downmodulating HIF-1α (Nadella et al., 2018). HIF-1α participates in the differentiation of immunosuppressive MDSCs into TAMs by enhancing the PD-L1 ligand, Arg1, and NOS2, actually serving as the significant intermediate to realize the poor immune status (Zhang J. et al., 2018; Wu et al., 2019). Apart from macrophage-mediated immunosuppression, the significant effect occurring in TAMs is pro-angiogenic by HIF stabilization. The significance of vascular growth for tumor growth and early metastasis is universally acknowledged. Hypoxia can upregulate the expression and release of VEGFA in TAMs *via* HIF-1α and its downstream effectors. The lack of macrophages or VEGFA expression can both remarkably reduce vascular density and tumor progression (Li S. et al., 2019). Additionally, hypoxic macrophage can produce the matrix metalloproteinase MMP9 to degrade thick and dense matrix in a HIF-1α-dependent way, facilitating the formation and extension of blood vessels in solid tumors (Riera-Domingo et al., 2020). More than influential on macrophages, the hypoxia state can alter multiple normal physiological processes of pancreatic tissue, thus fostering the environment beneficial to tumor progression, as diverse as the tumorigenesis, growth, immune evasion, metastasis, and so on.

The adjustment of glucose metabolism is simultaneously initiated under the hypoxic circumstance, defined as the Warburg effect, resulting in an accumulation of lactic acid in the tumor microenvironment (Lundahl et al., 2017). The differences in M1 and M2 macrophages lie in metabolic preferences: the energy supply of M1 macrophages is apt for glycolysis, whereas M2 macrophages are dependent on the TCA cycle. However, there is an inversion in the tumor microenvironment wherein M2 differentiation and glycolysis are equally predominant, seemingly contradictory to the above metabolic theory (Penny et al., 2016). There is an underlying mechanism deserving investigation to explain the disagreement under inflammation and tumor. Because of the uncoupling glycolysis and the TCA cycle, lactate acid acts as the primary circulating TCA substrate in most tissues and tumors, which is a distinctive approach of energy generation (Hui et al., 2017). The enriched lactate acid can inhibit various immune cell activities, contributing to the suppression of immune surveillance (Anderson et al., 2017; Dehne et al., 2017). The activation of the M2 phenotype is an indispensable link in the immunosuppressive tumor microenvironment, and high levels of lactate acid offer an impetus to TAMs toward M2 polarization (Ohashi et al., 2017; Zhang and Li, 2020). Nrf2 signaling is activated by lactate to skew the M2-like orientation in PDAC (Feng R. et al., 2018). There is an interesting feedback loop found by Ye et al. where CCL18 secreted by TAMs induces a glycolytic type in PDAC cells and, conversely, VCAM-1 produced by the tumor cells to mediate M2 deviation (Ye et al., 2018). On account of glucose metabolism having impacts on PDAC progression, recent related researches have proposed the anticancer role of metabolic regulation drugs, especially some diabetes medicines, such as metformin, which effectively alleviates the M2 polarization of TAMs, as well as reduces tumor cell invasion at normal but not high-glucose levels (Karnevi et al., 2014).

Furthermore, the TCA cycle as the primary metabolic pathway manages the transformation of multiple substances like sugars, fatty acids, nucleic acids, and proteins. The halt of the cycle leads to the accumulation and consumption of their components, creating the disbalance in metabolism, which affects the tumor microenvironment. Endogenous metabolites govern the specific procedure of inflammatory response, such as succinate regulating the IL-1β/HIF-1α inflammatory signaling axis. The amassment of succinate as the key part of the TCA cycle offers anti-inflammatory cues for immune cells like macrophages (Lampropoulou et al., 2016). In the tumor microenvironment, cancer cells secreted succinate to stimulate succinate receptor (SUCNR1) of TAMs, which actuates the macrophages to M2 tendency by triggering the PI3K–HIF-1α axis (Wu et al., 2020). Activated macrophages are commonly observed in the induction of itaconate, which has recently been reckoned as a regulator on macrophage metabolic reprogramming toward the anti-inflammatory type. Itaconate mitigates the oxidation of succinate by mediating succinate dehydrogenase, thereby leading to anti-inflammatory conversion *via* the accumulation of its succinate (Lampropoulou et al., 2016). Besides, itaconate can directly modify the protein KEAP1 *via* alkylation of cysteine residues, enabling Nrf2 to upregulate its downstream antioxidant and anti-inflammatory genes (Mills et al., 2018). The rise in itaconate production reprograms the phenotype of macrophages whether in tumor progression or inflammatory response. Glutaminolysis by αKG plays a significant role in M2 differentiation, involved in fatty acid oxidation and Jmjd3-dependent manner (Liu et al., 2017). Citrate accumulation is the most remarkable change in TCA cycle alteration and can take account for macrophage metabolic reprogramming, for which autologous nitric oxide (NO) is responsible. Apart from mitochondrial aconitase inhibited by macrophage-produced NO, macrophages render pyruvate away from pyruvate dehydrogenase under a NO-dependent and HIF-1α-independent manner, thereby promoting glutamine-based anaplerosis (Palmieri et al., 2020). Especially in tumor development, the change in metabolic conditions modulates the intracellular metabolic process and thus affects certain gene expression. Accumulative evidence leaves us to witness metabolism reprogramming not merely in macrophages but in all immune cells. Further explorations on metabolic reprogramming provide us with a new insight on macrophage polarization.

## Exosomes on TAM Polarization

Extracellular vesicles are often used as an important carrier of intercellular communication, shuttling between tumor cells and stromal cells of local or distant microenvironments. EVs are categorized according to their sizes and biogenesis mechanism, including exosomes, microvesicles, exosomes, apoptotic bodies, and oncosomes (Becker et al., 2016). Crucial message molecules enveloped by EVs are secreted into the microenvironment, which is discharged to regulate the gene expression of target cells following the recognition of the surface proteins and receptors on target cells. Notably, exosomes with diameters of less than 100 nm undertake the intermediate carrier to engage in cell communication, originally introduced by Johnstone et al. in 1987 at culturing sheep reticulocytes *in vitro* (Johnstone et al., 1987). Depending on exosomes’ transportation, tumor cells achieve cell-to-cell communication with macrophages, particularly affecting their phenotype. Exosomes from tumor cells package assorted proteins and chemokines with immunomodulatory capability, including CSF-1, CCL2, and TGF-β, to promote M2-like characterization of TAMs (Park et al., 2019). Multiple RNAs with regulatory functions including microRNAs, long non-coding RNAs, circular RNAs, and so on, are carried into target macrophages to enhance M2 feature gene expression (Chen X. et al., 2018; Hsieh et al., 2018; Wu et al., 2019). Furthermore, DAMPs as inflammation triggers are subject to exosome transportation, engaging in reprogramming TAMs. One of the DAMPs, KRAS^*G*12*D*^ protein, is induced by oxidative stress from tumor cells and enveloped into exosomes then captured by macrophages through an AGER-dependent mechanism, which orchestrates M2 differentiation through STAT3-dependent fatty acid oxidation (Dai et al., 2020). Previous findings demonstrate that proteins packaged into exosomes can maintain their activity following uptake by recipient cells. Membrane surface tyrosine kinase receptors traveled to macrophages as the fluxion of exosomes, such as the IL-6 receptor, highly enriched in exosomes from breast cancer cells, shaping macrophages toward alternatively activated phenotype by activating the STAT3 signaling of macrophages (Ham et al., 2018). Intrinsic compositions of exosomes have impacts on the intercellular cross talk. Linton et al. (2018) have observed that the exosomes from APC-1 (an ascites-derived metastatic pancreatic cancer cell line) exhibit a difference in composition with less metastatic PDAC cancer cell lines and normal pancreatic epithelial cell lines. Exosomes from APC-1 can overexpress the adhesion molecule ICAM-1 and fatty acid AA, which is conducive to their fusion faculty with target macrophages. Therefore, alterations in exosomes in content and composition have been taking place as the tumor progresses; hence, exosomes have a promising application of early detection. Glypican-1 (GPC1) as a cell membrane proteoglycan abundant in exosomes from cancer cells is capable of indicating the tumor burden and reflecting the prognosis of pre- and post-surgical patients, which can be applied into early detection of pancreatic cancer (Melo et al., 2015). Owing to their tropism of action, exosomes have the potential as therapeutic targets to be employed in targeted therapy of PDAC. Targeting the signaling vehicle, exosomes can effectively be a promising approach to convert TAMs’ adverse characteristics. By successful transfection of a plasmid with miR-155 and miR-125b to pancreatic cancer cells, M1-directed reprogramming of macrophages was elicited in the basement of altered exosome content leading to differential communication (Su et al., 2016). EZR, a member of the ERM family, in charge of cell proliferation, morphogenesis, migration, and adhesion and mediating plasma membrane signaling transduction, can modulate the polarization of macrophages to the M2 phenotype. However, the knockdown of EZR TAMs into the M1 phenotype provides a potential candidate to reprogram macrophages by modulating molecular pattern on EVs (Chang et al., 2020). Label molecules of exosomes are recognized by target cells and then initiate its related regulation toward the selected cells. Removal or masking of label molecules is a promising approach to remold the exosome-based communication, which remodels the immunophenotype and immune structure of the tumor microenvironment, beneficial to immunotherapy of PDAC. Simultaneously, exosomes are non-immunogenic nanosized vesicles that have received significant attention as an efficient drug delivery system. Since exosomes carry similar surface receptors of tumor cells and stromal cells such as immune cells and fibroblasts, they can be effectively recognized and uptaken by surrounding cells in tumor stroma. A comparative analysis parallelly compared the drug delivery efficiency of exosomes from pancreatic cancer cells, pancreatic stellate cells, and macrophages, showing that macrophage-derived exosomes have the highest activity of antitumor efficacy (Kanchanapally et al., 2019). In conclusion, exosomes have broad research prospects and may become useful drug transport materials and therapeutic targets in the future.

## Potential Targets to Regulate TAMs

**FIGURE 3:**
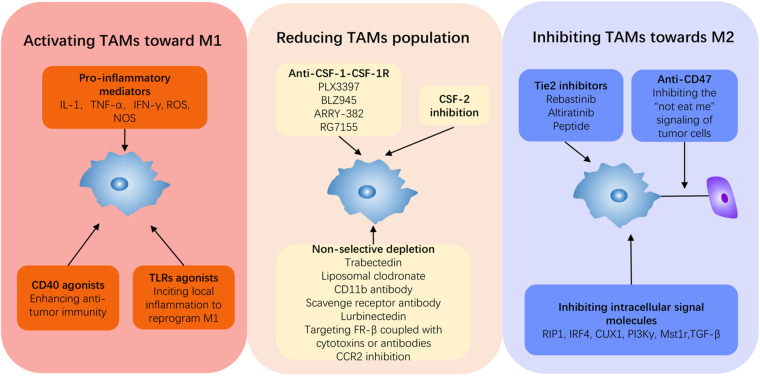
Current strategies to reprogram TAMs in PDAC. Induction from M2 to M1 is a fundamental concept for PDAC therapy, and it can be summarized into three approaches: activating TAMs to M1, inhibiting TAMs to M2, and depleting the monocyte–macrophage system. Immune promoters can be applied to activate the immune system to switch on M1 such as some pro-inflammatory cytokines, TLR agonists, and CD40 agonists. Anti-CSF-1, anti-Tie2, and anti-CD47 can all repress TAMs toward M2. Beyond these, inhibiting intercellular signal molecules can target and block M2 deviation, thus reviving antitumor activity.

With the development of deep research on the mononuclear macrophage system, conventional dualism has been advanced into macrophage activation theory, which provides us the accurate cognition of macrophage behavior in inflammation and tumor progression. Emerging advance on macrophage adjustment has been proposed, such as blockade of CSF-1/CSF-1R, CD40 agonists, and so on, which are favorable to re-educate TAMs to M1 from the M2 state (Table 1). An increasing number of approaches to target macrophages make it effective to repress tumor progression and present an excellent application in tumor treatment (Figure 3).

**TABLE 1:** Potential targets to reprogram TAMs polarization.

Potential targets	Therapeutic Effects	References
**Blocking CSF-1/CSF-1R signaling**		
CSF-1R inhibitors/CSF-1 neutralizing antibodies	Decreasing CD206Hi TAMs	Pradel et al., 2016; Ries et al., 2014
	Supporting anti-tumoral IFN responses and T cell activities	
	Overcoming immunosuppression	
	Enhancing antigen presentation	
Anti-CSF-1/CSF-1R/CTLA4 and PD1 antagonists	Enhancing responses to checkpoint immunotherapy	Zhu et al., 2014
	Promoting CD8 + T cells anti-tumoral activity	
Anti-CSF-1 + Radiation	Reducing TAMs recruitment after radiation	Jones et al., 2018
	Increasing the ratio of M1/M2	
	Promoting cytotoxic CD8^+^ T lymphocytes infiltration	
Anti-CSF-1R/Anti-PD-1/GVAX	Increases PD-1^+^CD137^+^ T lymphocytes and PD-1^+^OX40^+^CD4^+^ T lymphocytes in the tumor microenvironment	Saung et al., 2018
	Promoting expression of IFN-γ.	
**Activating CD40**		
CD40 agonist	Activating TCR signals and promoting T lymphocytes efficacy and increasing activity and longevity of T cells	Long et al., 2016; Stromnes et al., 2019
	Reprogramming TAMs to M1 and inhibiting M2 polarization	
	Promoting PDAC sensitivity to chemotherapy	
CD40 agonist/T cell–inducing vaccine/anti–PD-1	Achieving the optimal T cell activation	Ma et al., 2019
	Promotes the development of functional T cell memory	
	Reducing M2 macrophages and MDSCs while increasing M1 and DCs	
**Anti-Tie2+**		
Anti-Tie2 + /Anti-VEGFR2	Lessening the relapse of pancreatic tumor	Piao et al., 2016; Harney et al., 2017; Arai et al., 2019
	Reducing the accumulation of anti-angiogenesis therapy	
	Enhancing anti-angiogenesis therapy	
**Anti-CD-47**		
Anti-CD47	Improving macrophages to scavenge PDAC cells	Michaels et al., 2018; Pan et al., 2019
	Increasing the ratio of M1/M2	
	Reconstituting T cells in a more active direction	
**Activating Toll-like receptors**		
Activating TLR3, 4, 7, 8 and 9	Recalibrating the immune status of tumor cells and immune cells	Wanderley et al., 2018
	Promoting M1 polarization while inhibiting M2 deviation	
R848-loaded β-cyclodextrin nanoparticles	Activating the M1 phenotype by activating TLR7 and TLR8	Rodell et al., 2018
SC1	Promoting a systemic release of IFNα	Vascotto et al., 2019
	Resulting in the activation of circulating immune cells	
	Creating the circumstance apt to M1 polarization	
Phagocytosis-activating ligand/TLR agonist/anti-CD40 antibody	Achieving eighty percent recovery in the murine model of the aggressive pancreatic tumor	Caisová et al., 2018
Peptide-pulsed DCs/TLR3 agonist/poly-ICLC	Inducing a measurable tumor-specific T cell population in patients with advanced pancreatic cancer	Mehrotra et al., 2017
Quantum dot (QD) pulsed-DC vaccines/TLR9 agonists	Reversing macrophage polarization	Liu F. et al., 2020
	Boosting antigen-specific T-cell immunity	
	Eliciting a potent response to the innate and adaptive immune system	
	Breaking out the immunosuppressive barrier	
**Targeting the intercellular signaling molecules**		
Inhibiting RIP1	Enhancing the efficacy of the checkpoint receptor PD-1 and the costimulatory ligand ICOS based immunotherapies	Wang W. et al., 2018
	Reprogramming the state of macrophage toward an MHCII^hi^TNFα^+^IFNγ^+^ immunogenic phenotype	
Inhibiting IRF4	Creating the immune responsive environment upregulating the expression of IL-1α	Bastea et al., 2019
	Activating IFNγ^+^CD4^+^ and CD8^+^ T lymphocytes population	
Inhibiting CUX1	Reviving the activation of NF-κB signaling to drive the polarization of M1	Kühnemuth et al., 2015; Liu F. et al., 2020
Activating PI3Kγ	Reprogramming TAMs toward M1 tendency	Gunderson et al., 2016; Kaneda et al., 2016
	Abolishing CD8^+^ T-cell-mediated tumor suppression	
Inhibiting Mst1r	Lessening the accumulation of MRC1^+^Arg^+^ macrophages in the PDAC microenvironment	Babicky et al., 2019
	Transforming M1 polarization in the orthotopic model	
**Depletion and inhibition of recruitment of TAMs**		
Diphtheria toxin	Exhausting macrophages to establish the TAMs-deficient mice models in experiments	Zhang Y. et al., 2017
Trabectedin	Inducing the apoptosis of monocytes and macrophages via activating the caspase 8 apoptotic pathway	Germano et al., 2013; Borgoni et al., 2018
	Promoting infiltrating T cells anti-tumor phenotype	
Liposomal Clodronate	Eliminating CD11b + macrophages in the pancreas and other organs including liver, lung, and spleen	Griesmann et al., 2017
	Effectively hampering PDAC progression	
Lurbinectedin	Triggering caspase-dependent apoptosis of monocytes and macrophages via constituting lurbinectedin-DNA adducts	Céspedes et al., 2016
	Accomplishing TAMs depletion thus leading to cytidine deaminase downregulation in PDAC, alleviating the chemoresistance	
Folate Receptor β (FR-β)	Being a potential address label for transporting therapeutic pharmacological molecules into macrophages to exclusively attack macrophages to reduce their population	Hu et al., 2019
CCR2 inhibition + FOLFIRINOX	The local tumor control has been achieved in 32 of 33 patients (97%).	Nywening et al., 2016

*GVAX, the pancreatic cancer vaccine; R848-loaded β-cyclodextrin nanoparticles, TLR7 and TLR8 agonist; SC1, TLR7 agonist; RIP1, Receptor-interacting protein 1 (RIP1) kinase; FR-β, Folate receptor β.*

### Anti-CSF-1/CSF-1R

CSF-1/CSF-1R signaling has undergone relatively explicit investigation so far, the well-known fact that CSF-1 can attract, polarize, and sustain TAMs infiltrating in the tumor microenvironment, especially in PDAC with affluent stroma. The blockade of CSF-1/CSF-1R activation can be a promising approach in pancreatic cancer therapy *via* reducing the TAM population, using combinative partners of standard treatment and immunotherapeutic agents (Cannarile et al., 2017). The data from Zhu et al. (2014) have shown that inhibiting CSF-1R signaling depletes CD206^hi^ TAMs and reprograms the remaining macrophages to immune-active M1 type, paralleling with enhanced antitumor interferon, promoting T-cell infiltration, and preventing tumor progression. Depletion of TAMs by the CSF inhibitor has conspicuously enhanced the tumoricidal activity of radiation, indicative of its association with promoting adaptive immunity (Jones et al., 2018). However, CSF-1R inhibition may not deplete CD206^hi^ TAMs activated by IL-4 and GM-CSF, indicating that patients with IL-4 and GM-CSF overexpression may not benefit from CSF-1R blocking therapy (Pradel et al., 2016). Classical small molecule inhibitors of the CSF-1 pathway are PLX-3397, BLZ945, and ARRY-382, widely adopted in preclinical experimental researches of multiple tumors (Cannarile et al., 2017). The monoclonal CSF-1R antibody RG7155 blocks receptor dimerization, thereby improving objective clinical responses and increasing the CD8^+^ T-cell amounts (Ries et al., 2014). Another report from Candido et al. (2018) of the selective CSF-1R inhibitor AZD7507 in genetic PDAC model mice has underlined its profound antitumoral effect and T-cell activation, not subject to PD1 inhibition, particularly in the squamous subtype with abundant TAM infiltration. The reactivation of T cells is the core part, but overactivated immune checkpoint signals consume these T cells’ activity, gradually depriving the antitumor effect (Jones et al., 2018). Besides, growing recruitment of CXCR2^+^ neutrophils was incited by fibroblasts reactively overproducing corresponding chemokines from CSF-1R inhibition (Kumar et al., 2017). These adverse alterations create a big challenge to the administration of anti-CSF-1, and thus, shortcomings of monotherapy must be rectified in combination with other therapeutic methods. For instance, dual inhibition of PI3K-γ and CSF-1R by a nanomicelle encapsulating the PI3K-γ inhibitor BEZ 235 and CSF-1R-siRNA has been reported to largely diminish M2 macrophage infiltration and remove the immune blockade in the tumor microenvironment of PDAC (Li et al., 2020). A triple combination of anti-CSF-1R, anti-PD-1, and PDAC vaccine (GVAX) contributes to the conversion of exhausted PD-1^+^ T cells to CD137^+^ activated effector T cells, intriguing the vigorous antineoplastic immunity (Saung et al., 2018). Anti-CSF-1 is an effective adjuvant therapy applied to realistic practice and obtains a curative effect combined with other therapeutic regimens. On the basis of preclinical investigation, these classical and novel inhibitors have undergone clinical examinations to test their clinical therapeutic values. Nevertheless, some adverse effects of this method need to be overcome in future clinical applications.

### CD40 Agonists

The application of targeting CD40 in cancer immunotherapy recently can be foreseen as the development of tumor immunity research, through irritating Th1 immunity *via* maturing dendritic cells and driving M2 to M1. CD40 is a cell surface molecular receptor of the TNF receptor family and expressed on a wide range of leukocytes including monocytes/macrophages, B cells, and dendritic cells, being responsible for antitumor immunity activation. In PDAC, tumor cells are CD40-negative, whereas stromal cells express CD40, particularly TAMs. Progressively, CD40 activation has been thought to resort to macrophages to regress and counteract tumor. A study from Beatty’s group (Beatty et al., 2011) explores the efficacy of CD40 agonists in both humans and mice. Systemic CD40 activation abolished tumor-induced immunosuppression and incited T-cell-dependent antineoplastic immunity in PDAC. Interestingly, depletion of macrophages obscured the previous antitumor effect by CD40 agonists, which underlines the significance of macrophages in the application of CD40 activation. Despite acting as a T-cell activation pathway, CD40 activation does exert its antitumor mechanism by reactivating macrophages to change the immune status of the tumor microenvironment. The activation of CD40, along with the elevated production of IFNγ and CCL2, drives a subset of Ly6C^+^CCR2^+^ macrophage infiltration (Long et al., 2016), as well as upregulates MHC class II molecules and the co-stimulatory molecule CD86 (Beatty et al., 2011). Besides, a recent study has pointed out that agonistic anti-CD40 reprograms the TAM phenotype to antitumoral and accumulates intratumoral population as well as increases longevity of TCR-engineered T cells (Stromnes et al., 2019). A phase I study of 22 patients with chemotherapy-naive advanced PDAC revealed the excellent tolerance of CD40 agonist allied with gemcitabine, by reviving T-cell functions and reawaking the antitumor activity in patients with PDAC (Beatty et al., 2013). CD40 agonists augment antitumor immunity in PDAC in a non-redundant manner, which is effective in immune checkpoint blockade (Rech et al., 2018). The triple combination of T-cell-inducing vaccine, CD40 agonist, and anti-PD-1 significantly disarms the functionality of multiple suppressor cell populations by achieving the optimal T-cell activation, shown by an expansion of MHCII^hi^ CD86^hi^ CD11b^+^ F4/80^+^ and declines in the population of Arg1-expressed macrophages and immature MDSCs (Ma et al., 2019). The administration of CD40 agonists stimulates the immune state of the tumor site, and CD40-activated macrophages may promote the delivery of chemotherapy in the destructed stroma (Vonderheide, 2020). Visibly, the appearance of macrophages plays a significant role in CD40 activation treatment, but the specific mechanism of macrophages is worthy of further investigation.

### Tie2 Inhibitors

Tie2 is a tyrosine kinase receptor and Tie2-expressing macrophages have been generally reckoned as a predictive marker of poor prognosis in multiple cancers, attributed to the influence to angiogenesis, vascular remodeling in tumor entity, and macrophage differentiation (Matsubara et al., 2013; Mao et al., 2017; Yang et al., 2018). Tie2^+^ macrophage infiltration after chemotherapy indicates the possibility of tumor recurrence. Especially, TAMs with high expression of Tie2 aggregate around tumor blood vessels and are selectively activated into M2-like subpopulations following chemotherapy, which promote tumor blood vessel reconstruction and recurrence in a VEGFA-dependent manner (Hughes et al., 2015). The molecule marker Tie2 can be counted as a unique feature of M2-like macrophages, involved in angiogenesis *via* the ang2–Tie2 axis. Accordingly, the elimination of Tie2^+^ macrophages can acquire excellent therapeutic benefits. Rebastinib is a potent and selective inhibitor of the Tie2 receptor tyrosine kinase. The preclinical validation on the administration of rebastinib in breast carcinoma and pancreatic neuroendocrine tumor has revealed that rebastinib inhibits Ang2/Tie2 signaling *via* multiple pathways (Harney et al., 2017). Targeting Ang2/Tie2 not only suppresses the angiogenesis of Tie2^+^ macrophages but also modifies the immunosuppressive tumor microenvironment, which consolidates the antitumor therapy. Long-standing anti-VEGFA therapy leads to the upregulation of Ang2 production and enhanced infiltration by Tie2-expressing macrophages, which reduce the efficacy of targeting VEGFA in PDAC (Rigamonti et al., 2014). The dual blockade of VEGF and Tie2 significantly enhanced the prevention of tumor progression (Arai et al., 2019). Given that anti-Tie2 effectively complements the anti-angiogenesis of anti-VEGFA therapy, current strategies have focused on combining different strengths. Altiratinib, a novel balanced inhibitor of MET/TIE2/VEGFR2, exhibits a latent capacity of auxiliary treatment in glioblastoma, by virtue of their pleiotropic inhibitions among tumor growth, invasiveness, angiogenesis, and myeloid cell infiltration (Piao et al., 2016). A new peptide designed for ang2/VEGFR, a fusion protein of AS16 and IgG Fc fragment, can significantly reduce tumor volume, blood vessel density, and tumor-related macrophages (Zhu et al., 2018). Moreover, Zhang L. et al. (2019) designed a dual-responsive amphiphilic peptide to modify the small peptide T4 as a Tie2 inhibitor, endowing it with endurance in circulation and specifically targeting the tumor site. The nanoformulation can hamper the Tie2 signaling activation in Tie2^+^ macrophages and endothelial cells to exert its antitumor role. Thus, the Ang2/Tie2 axis is expected to be an effective therapeutic target of cancer immune therapy.

### Anti-CD47

As the first line of defense against pathogenic insults, macrophages can rapidly scavenge cell debris or pathogenic microorganism *via* an inflammation cascade. Dying cells secrete a biochemical agent, called a “find-me” signal, drawing phagocytes to their vicinity and being engulfed after recognition at the earliest stage of apoptosis, while the “not-eat-me” signals of healthy cells are identified to avoid phagocytosis. The mechanism is frequently hijacked by tumor cells to counteract phagocytosis, leading to the proliferation of tumor cells. Signal regulatory protein alpha (SIRPα)–CD47 is a classical molecule match to prohibit the phagocytosis of macrophages in tumor immune escape. Additionally, CD47-mediated protection against phagocytosis prolongs the retention of exosomes in the extracellular environment, favorable for tumor cells to modulate the tumor microenvironment (Kamerkar et al., 2017). In engulfment assays, blocking CD47 by a monoclonal antibody *in vitro* improves macrophages to scavenge PDAC cells, retarding metastatic progression and prolonging survival (Michaels et al., 2018). Anti-CD47 significantly alters the structure of TAMs in tumor stroma, with a rise in the ratio of M1/M2, simultaneously reconstituting T cells in a more active direction (Pan et al., 2019). Several microRNAs like miR-340 and miR128 in pancreatic cancer cells inversely correlated with CD47 expression and can negatively regulate it, which is a chance to be a molecular means of anti-CD47 (Xi et al., 2020a,b). Recently, Liu et al. have revealed the close relationship between the central carbon metabolism and the antitumor activity of macrophages, where alteration of the metabolic state in macrophages by the TLR9 agonist CpG induces phagocytosis to CD47-positive tumor cells (Liu et al., 2019). Targeting the CD47–SIRPα axis results in a functional skewing of macrophages toward an M1-like phenotype in tumor models, thus contributing to antitumor immune response, and it has promising clinical prospective in PDAC therapy.

### TLR Agonists

As we mentioned above, the inflammatory response is a double-edged sword in the development of pancreatic tumors. It is greatly convincing by adequate researches that inciting controlled and limited inflammatory response reactivates the immune system suppressed by the tumor microenvironment, thus enabling it to perform antitumor functions. Activation of TLR signaling using TLR agonists alters the immunosuppressive structure in the PDAC microenvironment, especially reorienting TAMs to induce immunogenic inflammation. Prakash et al. (2016) provided experimental evidence that TLR4 activation by LPS recalibrates the immune status of tumor cells and immune cells, particularly bringing antitumor potentials back to macrophages. The role of the classical antineoplastic agent paclitaxel has been broadened by the novel finding of reprogramming M2-polarized macrophages to M1-like phenotype *via* the TLR4 signaling pathway, apart from impeding the cell cycle (Wanderley et al., 2018). Similarly, activations of TLR3, TLR7, TLR8, and TLR9 prominently revive the innate immune cell functions in the tumor microenvironment, as potential immunologic adjuvants in cancer chemotherapy (Silva et al., 2015; Liu Z. et al., 2020). Related advancements put forward the availability of TLR reactivation in tumor therapy, but some tumor cell types are not sensitive to TLR agonist, just like mice bearing Pros1-expressed tumor do not show sensitiveness to TLR agonists (Ubil et al., 2018). Moreover, current compounds are not well tolerated during intravenous administration and restrained in disease conditions despite being suitable for topical use. Novel nanomaterials are introduced to improve drug delivery, strengthen efficacy, and reduce side effects. R848-loaded β-cyclodextrin nanoparticles offered efficient drug delivery to TAMs, as a potent driver of the M1 phenotype by activating TLR7 and TLR8 (Rodell et al., 2018). Likewise, TLR7/8 agonists effectually activated antitumoral inflammation by utilizing a nanoemulsion-based immunotherapeutic platform, steering the M2 toward the M1 phenotype and restoring the immunogenicity of the stromal environment (Kim et al., 2019). Vascotto et al. (2019) provided the preclinical characterization of SC1, a novel synthetic agonist with exquisite specificity for TLR7, and demonstrated that it induces a systemic release of IFNα, resulting in the activation of circulating immune cells and affording the circumstance apt for M1 polarization. Some natural compounds similar to TLR agonists are capable of reorienting immunosuppressive M2 to pro-inflammatory M1, such as cryptotanshinone acting on the TLR7/MyD88/NF-κB signaling pathway (Han et al., 2019) and the linear 3-O-methylated galactan WCCP-N-b targeting TLR2 signaling and suppressing STAT6 activation (Meng et al., 2019). Synergistic administration with TLR agonists has been adapted to conspicuously enhance both innate and adaptive immunity, comprising chemotherapy, immune checkpoint inhibitors, immune adjuvants, and radiotherapy. The alliance of phagocytosis-activating ligand, TLR agonist, and anti-CD40 antibody achieved 80% recovery in the murine model of aggressive pancreatic tumor (Caisová et al., 2018). TLR7/8 ligands along with radiotherapy contribute to a profound systemic antitumor immune reaction, coordinated by macrophages and dendritic cells and executed by NK and cytotoxic T cells (Schölch et al., 2015). It is worth noting that dendritic cell (DC) vaccines exhibit a great capacity for cancer immunotherapy, which is available for combination therapy of TLR agonists. A clinical trial verified the safety of vaccination with peptide-pulsed DCs in combination with the TLR3 agonist poly-ICLC, which induced a measurable tumor-specific T-cell population in patients with advanced pancreatic cancer (Mehrotra et al., 2017). Specifically, Liu F. et al. (2020) designed quantum dot (QD)-pulsed DC vaccines integrated with TLR9 agonists to reverse macrophage polarization, which elicited a potent response to the innate and adaptive immune system and ameliorated the immunosuppression of the tumor microenvironment. Taken together, TLR agonists significantly provide an expansive immune effect and systematically alter the immune state, particularly to intensify the tumoricidal activity of immune cells. The transformation of the tumor immune structure is the basis of the antitumor execution and the premise of immunoadjuvant therapy for tumors. A wide variety of TLR agonists have been designed to make this approach clinically possible, but the ambiguous relationship between inflammation and tumor remains an important factor affecting treatment outcomes, which is worthy of further discussion and investigation. However, it should be also noticed that aberrant long-term activation of TLRs in PDAC also could promote tumor growth and attenuate the efficacy of immunotherapy or conventional treatments.

### The Adjustments to Intracellular Signaling Molecules

Macrophage polarization is subject to the intricate regulation of multiple cellular signaling pathways. As researches further developed, the accurate grasp of polarization signaling pathways has transferred the therapeutic focus from extracellular ligand–receptor blockade to intracellular signaling regulation, more precisely, directly, and obviously. The inflammatory signaling pathways represented by NF-κB have a great influence on macrophage phenotype differentiation, owing to the release of various inflammatory cytokines depending on it. As was stated above, TLR-dependent inflammatory response triggers a new round of immune turbulence, which improves the immune capacity to eliminate tumor cells. The RIP1 is a serine/threonine-protein kinase, a putative master upstream regulator of TLR signaling, driving NF-κB and MAP kinase signaling in response to inflammatory stimuli. In TAMs, the persistent abnormal activation can lead to an M2-like differentiation, which mediated the immune tolerance in the tumor microenvironment. As shown by the study of Wang W. et al. (2018), RIP1 signaling in macrophages serves as a master regulator of immune tolerance in PDAC and the inhibition can enhance the efficacy of the checkpoint receptor PD-1 and the co-stimulatory ligand ICOS-based immunotherapies *via* reprogramming the state of macrophage toward an MHCII^hi^TNFα^+^IFNγ^+^ immunogenic phenotype in a STAT1-dependent manner. IRF4 is a crucial transcriptional factor for TAM alternative activation, while its inhibition creates the immune-responsive environment to combat immunosuppression and fibrosis in PDAC, with the upregulation of IL-1α and the activation of the population of IFNγ^+^CD4^+^ and CD8^+^ T lymphocytes (Bastea et al., 2019). Additionally, CUX1, the transcriptional factor suppressing M1 deviation, has been implicated to have the potential to regulate TAM polarization (Kühnemuth et al., 2015). The deprivation of oxygen and nutrients initiates metabolic reprogramming in TAMs, which involves the intersection molecule PI3Kγ. The blockade of PI3Kγ in PDAC-bearing mice reprograms TAMs to stimulate CD8^+^ T-cell-mediated tumor suppression and to inhibit tumor cell invasion, metastasis, and desmoplasia (Kaneda et al., 2016). The activation of B-cell signaling has also been discovered to engage in PI3Kγ-dependent M2 macrophage reprogramming and inhibiting BTK leads to M1 induction (Gunderson et al., 2016). Mst1r overexpression by KRAS aberration contributed to the increase of ADM and the acceleration of PanIN, as well as resulted in the accumulation of MRC1^+^Arg^+^ macrophages in the PDAC microenvironment. Certainly, the suppression of Mst1 expression transforms M1 polarization in the orthotopic model, providing further rationale for targeting Mst1r as a therapeutic strategy (Babicky et al., 2019). Manipulation of cellular signals makes it possible to precisely target tumors, but widespread suppression of certain signaling pathways may contribute to disorders in cellular biological processes. Various effective therapeutic targets have been identified, but specific reagents are still under development. Additionally, RNA vaccines and targeted drug delivery have promoted the clinical development of such drugs. Despite numerous restrictions to face, the therapeutic strategy can be progressed and enriched, with broad prospects and practical significance.

### Depletion and Inhibition of Recruitment of TAMs

Studies to date have illustrated the interrelationship between poor prognosis and amounts of TAMs infiltrating pancreatic tumor stroma. Except for orchestrating M2 toward M1, another therapeutic strategy is to diminish the circulating load of the mononuclear macrophage system by inhibiting cytogenesis or promoting cell death. Until now, the pharmacological depletion of TAMs has prevailed in experimental researches, but has not been clinically employed. Cytotoxic agents such as liposomal clodronate, trabectedin, and diphtheria toxin have been reported to systematically remove macrophages. Several studies have used diphtheria toxin to exhaust macrophages to establish TAM-deficient mice models, for the sake of investigating the role of TAMs in pancreatic cancer progression (Zhang Y. et al., 2017), but it lacks clinical values in cancer treatment. Trabectedin, a natural alkaloid derived initially from a Caribbean tunicate, with effective anticancer properties, has been approved for the therapy of ovarian cancer and soft-tissue sarcomas. Recent data have demonstrated that trabectedin induced the apoptosis of monocytes and macrophages in assorted surroundings by activating the caspase-8 apoptotic pathway (Germano et al., 2013). In the experimental research on pancreatic cancer, treatment with trabectedin significantly diminished the amounts of TAMs, which promote and trigger the infiltrating T-cell antitumor phenotype (Borgoni et al., 2018). However, the benefit of single-agent trabectedin is not significant and it is currently being studied in combination with other chemotherapeutic drugs. Liposomal clodronate is a common macrophage-depletion agent in pancreatic cancer experiment researches. The impact of liposomal clodronate is non-specific, which can eliminate CD11b^+^ macrophages in the pancreas and other organs including the liver, lung, and spleen (Griesmann et al., 2017). Like other macrophage-targeted agents, the effect is not obvious as a single administration. Additionally, specific antibodies against monocyte/macrophage surface molecules have the competence of TAM depletion. CD11b is a surface molecule universally expressed on the cell membrane of bone marrow-derived immune cells. CD11b^+^ macrophages are dominant in pancreatic cancer stroma, and the neutralized antibody anti-CD11b can effectively reduce TAM infiltration, which presents a better outcome (Chen C. C. et al., 2018). Antibodies scavenging receptors have demonstrated their counteraction of pancreatic cancer, further indicating the great potential targeting the TAM population (Neyen et al., 2013). Lurbinectedin (PM01183) is a novel antitumor agent with a broad panel of antitumor activity, including lung, ovarian, colorectal, and gastric carcinoma xenograft, which constitutes lurbinectedin–DNA adducts to eventually trigger caspase-dependent apoptosis. Its antitumor activity correlates with the depletion of TAMs in different tumor models. In the combination treatment of lurbinectedin and gemcitabine, lurbinectedin significantly accomplished TAM depletion thus leading to cytidine deaminase downregulation in PDAC, which aggravates the damage strength by gemcitabine (Céspedes et al., 2016). FR-β is exclusively overexpressed by TAMs in any kind of cancer entity. The FR-β^+^ macrophages infiltrating perivascular regions were observed in the tumor tissues of PDAC patients, explicitly correlating with tumor angiogenesis and high incidence of metastasis (Kurahara et al., 2012). FR-β has been perceived as a potential address label for transporting therapeutic pharmacological molecules into macrophages. Targeting FR-β coupled with cellular cytotoxins or antibodies can exclusively attack macrophages to reduce their population (Hu et al., 2019). Reducing the macrophage population can alleviate the accelerative influence of TAMs on tumor progression, being a favorable strategy allied with other therapeutic approaches. The realistic efficacy has been demonstrated by a recent study on the union of radiation, macrophage depletion, and anti-PD-L1 (Jones et al., 2018). Indeed, the clinical significance of reducing the TAM population has been enlightened in clinical application. A phase 1b study has verified the effectiveness and safety of the combination of targeting TAMs by CCR2 inhibition and FOLFIRINOX in locally advanced and borderline resectable pancreatic cancer. Eventually, imaging results have demonstrated local tumor control achieved in 32 of 33 patients (97%), along with less side effects (Nywening et al., 2016). Accordingly, targeting TAMs obviously facilitates the antitumor effect of chemotherapy, which can be developed into a potent therapeutic method.

## Discussion and Future Perspective

Tumor-associated macrophages emerge to have multiple functions from the initiation phase to the mature stage of PDAC, including tumorigenesis, immune evasion, metastasis, and chemoresistance. Current researches have emphasized TAM polarization defined as an educated phenotype of macrophages in line with surrounding immune conditions, almost accepted by all modulations from various environmental factors in PDAC stroma. There is no denying that extensive cross talk has been constructed among stromal cells. Various signal molecules constitute an intricate network to collectively engage in the construction of TAM differentiation, which establishes a stable and substantial foundation for supporting tumor progression. In other words, others can substitute the blockade in one pathway, which makes it in a dilemma to break the steady state of the tumor microenvironment. Therefore, therapeutic effects are attenuated in response to targeting alone macrophage treatment by enhancing other aspects, such as augmented immune checkpoints and increased tumor-associated neutrophils (Zhu et al., 2014). Current views advocate combination therapy, for instance, in alliance with anti-PD1/PD-L1. Reprogramming the M2 phenotype of TAMs can effectively alter the immune state of the tumor microenvironment and revive the antitumor activity of the immune system. Compared to non-selective depletion of macrophages, re-education of TAMs to antitumoral orientation not only restores their immune pattern but also destroys immunosuppression of the PDAC tumor microenvironment, equal to internally disintegrate the network constituted by tumor cells and stromal cells. However, the present approaches to promote TAMs toward M1 have some limitations, which due to the complicated regulated network, repolarization can be counteracted by underlying mechanisms worthy of further investigations. M2 orientation of TAMs is endowed by overall characteristics of the tumor microenvironment, with involvements of multiple pathways, and reprogramming TAMs must depend on the integrated alterations of the tumor microenvironment. Nonetheless, a new tumor microenvironment will be reinstituted with the restabilization of the environment, which renders it to eliminate tumor cells as thoroughly as possible. Tumor cells create a cradle advantageous to their growth and regulating tumor microenvironments is just to exorcise the shelter to effectively attack against tumor cells. As it is known to all, TAMs shoulder the chief responsibility of establishing the tumor microenvironment, thus targeting them to acquire radical alterations. Hence, it is greatly convincing that targeting TAM therapy has promising development prospects. Furthermore, recent advancement in nanotechnology has persistently updated nanotargeted drug delivery systems, which has promoted directed TAM treatment more effectively and precisely (Yang et al., 2020). The introduction of new nanomaterials in pancreatic cancer therapy has promoted traditional drug delivery and pharmacological efficacy. Especially, vesicle transport as the major intercellular communication approach can be better applied to improve the conversion rate of TAMs, superior to single antibody or receptor blockade, being the future development direction.

Taken together, the polarization of TAMs acts as a key link in the tumor microenvironment and exerts significant roles in PDAC progression, but more underlying mechanisms require further explorations. More comprehensive immunotherapeutic modes are hopefully developed to participate in PDAC treatment, and TAMs are a potential target to fundamentally overthrow the immunosuppressive constitution in pancreatic cancer. Reprogramming TAMs to recalibrate tumor immunity has a better prospect of clinic therapy in the future.

## Author Contributions

QL provided significant guidance for the review. SY and QFL drafted the manuscript and illustrated the figures for the manuscript. All authors approved the final manuscript.

## Conflict of Interest

The authors declare that the research was conducted in the absence of any commercial or financial relationships that could be construed as a potential conflict of interest.

## Abbreviations

ADCC: antibody-dependent cell-mediated cytotoxicity
ADM: acinar-to-ductal metaplasia
Arg1: arginase-1
α KG: α -ketoglutarate
BMDMs: bone marrow-derived macrophages
CCL: chemokine (C–C motif) ligand
CSFs: colony-stimulating factors
CTGF: connective tissue growth factor
DAMPs: damage-associated molecular patterns
EGFR: epidermal growth factor receptor
EMT: epithelial–mesenchymal transition
ERM: ezrin–radixin–moesin
ET: endothelin
ETAR: endothelin receptor A
ETBR: endothelin receptor B
EVs: extracellular vesicles
FR- β: folate receptor β
HGBM1: high mobility group box 1
HIF: hypoxia-inducible factors
HSCs: hematopoietic stem cells
IRF4: interferon regulatory factor 4
MDSCs: myeloid-derived suppressor cells
MIP-3 α: macrophage inflammatory protein-3 α
MMPs: matrix metalloproteinases
NACRT: neoadjuvant chemoradiotherapy
NK: natural killer
NNK: nitrosamine-4-(methylnitrosamino)-1-(3-pyridyl)-1-butanone
NSAIDs: non-steroidal anti-inflammatory drugs
PanIN: pancreatic intraepithelial neoplasia
PDAC: pancreatic ductal adenocarcinoma
PDPK1: 3-phosphoinositide-dependent protein kinase 1
PGE: prostaglandin E
PGK1: phosphoglycerate kinase 1
PRRs: pattern recognition receptors
RIP1: receptor-interacting serine/threonine-protein kinase 1
SUCNR1: succinate receptor 1
TAMs: tumor-associated macrophages
TCA cycle: tricarboxylic acid cycle
TREM-1: triggering receptor expressed on myeloid cells-1.
